# Evaluation of healing at molar extraction sites with ridge preservation using a non‐resorbable dense polytetrafluoroethylene membrane: A four‐arm cohort prospective study

**DOI:** 10.1002/cre2.459

**Published:** 2021-06-06

**Authors:** Arwa M. Al Hugail, Brian L. Mealey, Christopher Walker, Shaimaa Al Harthi, Mylinh Duong, Marcel Noujeim, David J. Lasho, Thomas J. Prihoda, Guy Huynh‐Ba

**Affiliations:** ^1^ Department of Periodontics UT Health San Antonio San Antonio Texas USA; ^2^ Taif University Saudi Arabia; ^3^ Private Consultant, Department of Comprehensive Dentistry San Antonio Texas USA; ^4^ Department of Pathology UT Health San Antonio San Antonio Texas USA; ^5^ Private Practice Seattle Washington USA

**Keywords:** alveolar bone grafting, bone resorption, dental implants, tooth extraction

## Abstract

**Objectives:**

To examine ridge dimensional changes and histologic parameters of healing when ridge preservation (RP) was performed at molar sites using dense polytetrafluoroethylene (dPTFE) membrane alone, without a bone graft.

**Material and Methods:**

Eighteen patients had molar extraction and RP using dPTFE membrane alone. Ridge dimensions were measured using two standardized cone beam computerized tomography (CBCT) scans taken within 72 h and 3 months following extraction. Following a 3‐month healing period, an implant osteotomy was prepared using a trephine drill and bone cores were collected for histological analysis. Four‐arm analyses were performed using data from three previously published study arms of the same research group.

**Results:**

There was a significant change in the buccal ridge height between the four groups at all aspects of the socket. Alveolar ridge width reduction at 3 mm from crest for all aspects (mesial, midpoint, distal) of the socket showed statistically significant difference for dPTFE alone group compared to the other three groups. Percentage of vital bone formation (62.10%) was significantly greater in dPTFE alone group compared to the other groups.

**Conclusions:**

RP using dPTFE membrane alone in molar sites with intact socket walls showed successful outcomes in maintaining ridge dimensions and in histologic wound healing.

## 1 INTRODUCTION

Dimensional changes of the alveolar ridge following natural healing of tooth extraction sites are well documented in the literature (Amler et al., 1960; Pietrokovski & Massler, 1967). Schropp et al. (2003) showed that 50% of the ridge width was lost during the first year, with two thirds of that loss taking place during the first 3 months following extraction. A systematic review reported a mean ridge width reduction of 3.87 mm and mid‐buccal height loss of 1.67 mm post extraction in sockets left to heal naturally (van der Weijden et al., 2009).

Unfavorable dimensional changes resulting from this healing process may necessitate technique sensitive guided bone regeneration (GBR) procedures prior to dental implant placement. Ridge preservation procedures (RP) may limit these dimensional changes (Araújo & Lindhe, 2009; Darby et al., 2009; Iasella et al., 2003; Vignoletti et al., 2012). A systematic review evaluating the effect of RP following extraction of non‐molar teeth showed a mean reduction of 1.89 mm in buccolingual width and 2.07 mm in buccal height, which was significantly less than natural healing in the control group (Avila‐Ortiz et al., 2014). Another study found that 58% of non‐preserved premolar and molar extraction sites required bone grafting at time of implant placement compared to only 7% of the sites having RP (Cardaropoli et al., 2015). To date, no one specific biomaterial or technique for RP has been shown to be superior (Atieh et al., 2015; Corbella et al., 2017). The use of a dPTFE membrane over the socket of an extracted tooth without bone graft material has been shown to be clinically and histologically effective as RP technique when the membrane was left to heal exposed (Hoffmann et al., 2008). However, there is limited evidence examining the use of a non‐resorbable membrane with no bone graft as RP technique. Therefore the primary purpose of this study is to examine soft and hard tissue dimensional changes and the histologic parameters of healing when RP was performed at molar extraction sites using a dPTFE membrane alone. Secondary objectives were to compare these dimensional changes to the results in three previously published study arms of molar RP from the same research group, including: (a) No RP (control; Walker et al., 2017); (b) RP using mineralized freeze‐dried bone allograft (FDBA) with dPTFE membrane (test 1; Walker et al., 2017), and (c) RP using FDBA with collagen wound dressing (CWD; test 2; Al Harthi et al., 2019).

In addition, histologic outcomes of wound healing for the current study arm were compared to the same histologic parameters in the abovementioned treatment arms (Duong et al., 2019).

## 2 MATERIALS AND METHODS

This study originated as a randomized controlled clinical trial (RCT) comparing natural healing without RP to RP using FDBA with dPTFE membrane at molar extraction sites (Walker et al., 2017). Registered under Clinicaltrials.gov ID: NCT02543398). Consecutive to the completion of the RCT, the authors enrolled patients in a new study arm that examined RP at molar sites using FDBA with an overlying CWD (Al Harthi et al., 2019). The histologic parameters of healing including the percentage of vital bone, residual graft (when applicable), and connective tissue/other (CT/other) for bone cores obtained from the abovementioned study arms were then reported in a separate paper (Duong et al., 2019). Therefore, the current study is best described as a four‐arm cohort prospective study.

This study was conducted in accordance with the Helsinki Declaration of 1975, as revised in 2013. The protocol was approved by the institutional review board of UT Health San Antonio (Protocol number: HSC20170339H). Written informed consent was obtained for each patient by the Graduate Periodontics research team.

### 2.1 Participant enrollment protocol

One hundred and six patients, 27 in the present study and 79 from the previous three arms (Al Harthi et al., 2019; Walker et al., 2017), referred to the Graduate Periodontics department at the UT Health School of Dentistry, San Antonio, Texas who required extraction of a single molar due to non‐restorable carious lesion, prosthetic or endodontic failure, or root fracture followed by replacement with a dental implant were enrolled between January 2014 to November 2018.

Participants were considered eligible if they had ≥10 mm of radiographic alveolar bone height, without impinging on the maxillary sinus or inferior alveolar canal and adequate restorative space for dental implant‐retained restoration. Exclusion criteria included: (a) active localized or systemic infection and untreated periodontal diseases; (b) pregnant women or intended to become pregnant during the study period; (c) medical conditions or taking medications that may negatively affect soft and hard tissue healing; (d) smoking more than 10 cigarettes per day; (e) root of the tooth to be extracted was not in alignment with proposed implant osteotomy; and (f) more than 50% dehiscence of the total socket depth on either buccal or lingual wall at time of extraction.

### 2.2 Surgical protocol

Following enrollment, alginate impressions were made for fabrication of a customized stent (Essix ACE plastic 0.04″ [1 mm] thickness, DENTSPLY, York, PA) with a radiographic fiducial marker (US Patent 20060241406 A1, Dr. Noujeim, UT Health, San Antonio, TX) to be used at the time of CBCT scans as described by Walker et al. (2017)). On the day of extraction, keratinized tissue (KT) width for mandibular molars was measured to the nearest 0.5 mm using a periodontal probe (UNC‐15, G. Hartzell & Son, Concord, CA) from the gingival margin to the mucogingival junction (MGJ) at the mid‐buccal and mid‐lingual sites of the molar to be extracted. Baseline KT was calculated by adding the width of the socket recorded immediately following extraction at the middle of the socket to the preoperative buccal and lingual KT width. After obtaining anesthesia, full‐thickness flaps were reflected not to exceed 3 mm from the crest, and a minimally traumatic molar extraction was performed. Following extraction, the integrity of the socket was evaluated for the presence of dehiscence or fenestration. If a dehiscence greater than 50% of the socket depth was present, the patient was exited from the study and standard care therapy was delivered. The inter‐radicular bone, if present, was removed to a level at least 6 mm apical to the bony crest to prevent collecting native bone at the time of bone core harvest.

The socket was covered with a dPTFE membrane (Cytoplast TXT‐200 Single, Osteogenics Biomedical, Lubbock, TX) extending 3 mm beyond the buccal and lingual bony crest. No bone graft was used. Flaps were approximated and sutured together using 4–0 PTFE sutures (Cytoplast PTFE sutures, Osteogenics Biomedical, Lubbock, TX) with no attempt for primary closure. A CBCT scan (3DX Accuitomo, J. Morita USA, Irvine, CA) was taken with the radiographic stent in place within 72 h of extraction.

All patients were prescribed amoxicillin 500 mg or clindamycin 300 mg three times a day for a week. A 0.12% chlorhexidine gluconate mouth rinse was used twice a day for 4 weeks following extraction. First postoperative visit was scheduled at 14 days post extraction followed by a second visit for removal of non‐resorbable sutures and membrane between 21 and 28 days postoperatively.

Three months following extraction, participants returned for the second CBCT (final) taken with the same radiographic stent. Within 3 weeks from the final CBCT, implant surgery was scheduled. On the day of implant placement surgery, the width of KT was measured at the prospective mandibular implant sites by using a piece of floss placed passively over the KT of the healed ridge and marked at the MGJ on both the buccal and lingual aspects, then measured to the nearest 0.5 mm with the periodontal probe. Full thickness flaps were reflected, and the implant osteotomy initiated with a trephine drill (Salvin Dental Specialties, Charlotte, NC) to allow retrieval of a bone core of at least 6 mm in length and 2 mm in diameter at the middle of the former socket. The harvested bone core was immediately placed in 10% formalin. Thereafter, implant osteotomy was continued with consecutive drilling using the implant manufacturer's recommendations. A dental implant with a diameter of 4.7 mm (Zimmer Biomet, Warsaw, IN) or 4.8 mm (Straumann, Basel, Switzerland) was placed. In cases where there was implant surface exposure >4 mm^2^ (2 x 2 mm), bone grafting at time of implant placement was performed and recorded. A cover screw or healing abutment was attached based on the surgeon's discretion. Following implant placement, patients completed the study and standard care was provided thereafter.

### 2.3 Radiographic measurement protocol

Two CBCT scans were taken for each patient from September 2017 to February 2019 at the UT Health San Antonio, Oral and Maxillofacial Radiology clinic. Methodology of the radiographic measurement technique was described in detail previously (Walker et al., 2017).

### 2.4 Histologic processing and analysis

The histologic processing and histomorphometric analysis of harvested bone cores followed the same protocol previously described by Beck and Mealey (2010). Briefly, the processed core was viewed under ×4 microscope magnification and digital pictures of the core were captured. The pictures were then exported to imaging software (Adobe Photoshop CC 20.0.2, Adobe, San Jose, CA) and merged into one image. Vital bone and CT/other were identified and traced separately. Individual images of each tissue component were generated and converted into binary images (Image J, National Institutes of Health, Bethesda, MD) to calculate the total pixels in each image. The percentage of total area of each tissue component was then calculated based on the total number of pixels.

### 2.5 Statistical analysis

Based on the results reported by two previous studies (Leblebicioglu et al., 2013; van der Weijden et al., 2009) the distribution of bone width changes was expected to be normally distributed with a mean of −1.21 mm and a standard deviation (*SD*) of 0.54 mm when no RP is performed. We assumed that a clinically significant effect of RP will be reducing the loss of ridge width by 1 mm, resulting in a mean of 0.21 mm in the treated group.

Eight subjects in each group would result in a power of 0.90 to detect such a difference in width using a Student's *t* test for independent samples in a two‐tailed test with alpha = 0.05. The mean crestal bone height change reported by van der Weijden et al. (2009) was −1.53 mm with a *SD* of 0.88 mm. If a difference of 1 mm is again used as clinically important, 18 subjects per group resulted in a power of 0.90 to detect such a difference in ridge height using a Student's *t* test for independent samples in a two‐tailed test with alpha = 0.05. Use of a larger number of 18 subjects per group thus resulted in sufficient power for both outcomes. It was anticipated that approximately 70% of patients would complete the study; thus, the planned enrollment was 26 subjects. If the true population mean difference is 1 mm or more and the population standard deviations are as above, then the proposed sample is sufficient to detect a clinically significant bone width change mean difference and crestal height changes between patients who received ridge preservation with a dPTFE membrane (test 3) compared to no RP (control) and other RP techniques (test 1, test 2) reported previously by the same research group (Al Harthi et al., 2019; Walker et al., 2017).

Analysis of variance comparing the four groups was done for each outcome measured as well as the computed change scores. This was followed with pairwise multiple comparison *t* tests where *p*‐values less than 0.05 were reported. Means and standard deviations are given for descriptive statistics on these analyses. Residuals from the analysis of variance were plotted to help assure a bell shaped distribution approximating a normal distribution was present for a valid analysis.

Frequency data on demographics, molar and bone type distribution data were compared for the four groups with the exact chi‐square test. Intraclass correlation coefficients (ICC) were computed with variances for raters and subjects compared using restricted maximum likelihood method to describe rater reliability. The relationship between ridge width change and buccal plate thickness was analyzed with Pearson's correlation coefficient. All analyses were done using statistical software (SAS Version 9.4 for Windows, SAS Institute, Cary, NC). Intraexaminer agreement for radiographic measurement was determined by repeating 10% of the previous radiographic measurements performed by the research primary investigator (A.M.A.).

## 3 RESULTS

Eighteen of 27 enrolled patients, 8 females and 10 males with a mean age of 52.8 ± 16.1 years, completed the study. Nine patients were exited due to the following reasons: one patient decided not to have an implant, one patient did not get the first CBCT scan within the first 72 h following extraction, two patients withdrew from the study due to financial reasons, and five patients had greater than 50% buccal plate dehiscence. One core biopsy could not be harvested because of poor tissue quality; however, the implant was placed with good primary stability. Therefore, a total of 17 bone cores were harvested. None of the enrolled patients smoked or reported a history of uncontrolled systemic disease. Three patients required grafting at time of implant placement due to exposure of >4 mm^2^ of the implant surface. One patient did not receive an implant after successful bone core harvest due to lack of primary stability at time of implant placement. The site was grafted with bone allograft and implant placement was performed 3 months following graft healing. The remaining 17 patients had implants placed without any complications encountered during the 3 months healing period following implant placement and were referred to their restorative dentists thereafter.

Clinical, radiographic and histologic analyses were performed for the four treatment arms, which included a total of 79 patients, all treated by the same research group. From a previous RCT (Walker et al., 2017), 20 molar sites received a CWD sponge (CollaPlug, Zimmer Dental, Warsaw, IN) alone (Control) and 20 received RP using FDBA (enCore, Osteogenics Biomedical, Lubbock, TX) with a dPTFE membrane (Test 1). From a previous a case series (Al Harthi et al., 2019), 21 molar sites received RP using FDBA with an overlying CWD. The current study included 18 sites receiving dPTFE membrane alone. Differences in demographic characteristics (age and gender) and molar type distribution between the four groups (Table 1) were not statistically significant (*p* > 0.05). There was no significant difference in the KT change among the groups (*p* > 0.05; Table 2).

**TABLE 1:** Patient demographics and molar type distribution

Group		Patient age (years; mean ± *SD*)	Gender		Molar type			
			Male	Female	MX1	MD1	MX2	MD2
Walker et al. (2017)	Control (no RP)	53.3 ± 10.7	7	13	3	13	0	4
	Test 1 (FDBA + dPTFE)	54.1 ± 11.6	7	13	2	16	0	2
Al Harthi et al. (2019)	Test 2 (FDBA + CWD)	49.8 ± 14.6	7	14	2	15	0	4
Present study	Test 3 (dPTFE alone)	52.8 ± 16.1	10	8	3	12	0	3

Abbreviations: MX1, maxillary 1st molar; MX2, maxillary 2nd molar; MD1, mandibular 1st molar; MD2, mandibular 2nd molar.

**TABLE 2:** Keratinized tissue width change for mandibular molars

Group		Pre KT	Post KT	Loss of KT width
Walker et al. (2017)	Control (*n* = 17)	19.9 ± 3.7	12.1 ± 5.1	6.8 ± 3.0
	Test 1 (*n* = 18)	18.9 ± 3.6	13.1 ± 5.4	5.2 ± 3.7
Al Harthi et al. (2019)	Test 2 (*n* = 19)	22.1 ± 3.9	17.2 ± 3.4	4.9 ± 2.7
Present study	Test 3 (*n* = 15)	21.9 ± 3.9	15.8 ± 4.4	7.1 ± 5.0

*Note*: Keratinized tissue at baseline (Pre KT), 3 months after extraction (Post KT) and loss of width. All values expressed in mm (mean ± *SD*). No significant differences in change in KT among groups.

### 3.1 Radiographic observations

The time between CBCT scans was 94.5 ± 6.6, 95.1 ± 8.5, 89.4 ± 5.0, and 90.2 ± 5.0 days for control, test 1, test 2, and test 3, respectively. While there was a statistically significant difference between groups (*p* < 0.05) in the number of days between CBCTs, the maximum between group difference in the mean number of days was less than 6 days.

Baseline buccal and lingual plate thickness is seen in Table 3. At the mesial aspect of the socket the baseline buccal plate thickness was significantly greater in test 3 group compared to the other groups at 1, 3, and 5 mm from the bony crest. This was also true for the distal aspect of the socket at 1 and 3 mm from the bony crest. Statistically significant differences among the groups in the mean change of buccal ridge height were seen between the groups at all aspects of the socket (*p* < 0.05; Table 4). The greatest loss of ridge height at buccal measurement points was in the control group (no RP), with no differences among the three groups receiving RP. The average loss of lingual ridge height between the four groups was 0.6–1.0 mm, with no significant difference among the groups (*p* > 0.05; data not shown).

**TABLE 3:** Baseline buccal and lingual plate thickness

Group		Buccal plate thickness (mm)						Lingual plate thickness (mm)					
		Mesial			Distal			Mesial			Distal		
		1 mm	3 mm	5 mm	1 mm	3 mm	5 mm	1 mm	3 mm	5 mm	1 mm	3 mm	5 mm
Walker et al. (2017)	Control (no RP)	0.71 ±0.32^*^	1.01 ± 0.52^§^	1.52± 0.86^**^	1.16± 0.78^*^	1.82± 1.16^§^	2.55± 1.51^**^	1.98 ±1.18	3.30 ±1.48	4.04± 1.45^*^	2.24 ±1.50^*^	3.50± 1.60^†,‡^	4.10± 1.45^‖,¶^
	Test 1 (FDBA+ dPTFE)	0.84± 0.37^†,‡^	1.12± 0.54^‖^	1.57± 1.06^††^	1.07± 0.67^†^	1.74± 1.31^‖^	2.50± 1.87^††^	1.70 ±0.72	2.88 ±1.11	3.57± 1.43	1.50 ±0.76	2.31± 1.30^†^	2.81± 1.42^‖,††^
Al Harthi et al. (2019)	Test 2 (FDBA + CWD)	0.31± 0.52^‡,†^	0.56± 0.83^¶^	0.87± 1.14^‡‡^	0.33± 0.48^*,†,‡^	0.93± 0.83^¶^	1.33± 1.11^**,††,‡‡^	1.70 ±1.80	2.25 ±1.90	2.50± 1.85^*,†^	1.43 ±1.24^*^	2.06± 1.44^‡,§^	2.11± 1.41^¶,**^
Present study	Test 3 (dPTFE alone)	1.57±1.42^*,†,‡^	2.58± 1.81^§,‖,¶^	3.05± 1.78^**,††,‡‡^	1.98± 1.61^*,†,‡^	2.75± 2.18^§,‖,¶^	3.31± 2.17^‡‡^	2.05 ±1.86	3.14 ±2.18	4.03± 2.25^†^	1.91 ±1.33	3.27± 1.85^§^	4.06 ±2.04^**,††^

*Note*: Radiographic baseline buccal and lingual plate thickness measured at 1, 3, and 5 mm from alveolar crest expressed in mm (mean ± *SD*). ^*,†,‡,§,‖,¶,**,††,‡‡^: Similar superscript within a given column indicates a statistically significant difference (*p* < 0.05) between the groups at that measurement point.

**TABLE 4:** Buccal alveolar ridge height change

Group		Mean buccal height change (mm)		
		Mesiobuccal	Midbuccal	Distobuccal
Walker et al. (2017)	Control (no RP)	−3.02 ± 2.24^*,†^	−2.60 ± 2.06^‡,§^	−2.34 ± 1.72^‖^
	Test 1 (FDBA + dPTFE)	−1.11 ± 1.69^*^	−1.12 ± 1.60^‡^	−1.01 ± 1.85^‖^
Al Harthi et al. (2019)	Test 2 (FDBA + CWD)	−1.94 ± 1.73	−1.55 ± 0.93^§^	−1.30 ± 1.47
Present study	Test 3 (dPTFE alone)	−0.92 ± 2.08^†^	−1.60 ± 1.70	−1.65 ± 1.97

*Note*: Radiographic buccal ridge height changes expressed in mm (mean ± *SD*). ^*,†,‡,§,‖^: Similar superscript within a given column indicates a statistically significant difference (*p* < 0.05) between the groups at that measurement point.

The change in ridge width at 3, 5, and 7 mm from the bony crest is presented in Table 5. At 3 mm from the alveolar crest, the loss of ridge width was significantly smaller in test 3 than in the other groups at all aspects of the socket (mesial, midsocket, distal; *p* < 0.05). At 5 mm from the crest at both the mesial and midsocket aspects of the socket, a statistically significant difference was seen between the control and test three groups, and between test 1 and test 3 groups; while the distal aspect of the socket showed a significant difference only between the control and test 3 groups (*p* < 0.05). At 7 mm from the crest the only significant difference was observed at the mesial aspect between the control and test three groups (*p* < 0.05), with no significant difference at the mid and distal aspects of the socket between the groups (*p* > 0.05; Table 5).

**TABLE 5:** Alveolar ridge width change

Group		Ridge width change (mm)								
		Mesial			Midsocket			Distal		
		3 mm	5 mm	7 mm	3 mm	5 mm	7 mm	3 mm	5 mm	7 mm
Walker et al. (2017)	Control (no RP)	−2.97 ± 3.26^*^	−1.46 ± 2.34^§^	−0.73 ± 1.53^¶^	−3.11 ± 3.83^*^	−1.59 ± 2.23^§^	−0.53 ± 0.92	−2.52 ± 2.22^*,‡^	−1.07 ± 1.32^§^	−0.49 ± 0.79
	Test 1 (FDBA+ dPTFE)	−2.12 ± 2.43^†^	−1.00 ± 1.40^‖^	−0.53 ± 0.83	−2.48 ± 2.86^†^	−1.16 ± 1.97^‖^	−0.55 ± 1.29	−2.10 ± 2.53^†^	−0.70 ± 1.77	−0.22 ± 0.61
Al Harthi et al. (2019)	Test 2 (FDBA + CWD)	−1.46 ± 1.48^‡^	−0.72 ± 0.80	−0.15 ± 0.79	−1.64 ± 1.10^‡^	−0.79 ± 0.57	−0.40 ± 0.45	−1.12 ± 1.07^‡^	−0.47 ± 0.61	−0.20 ± 0.42
Present study	Test 3 (dPTFE alone)	−0.60± 0.86^*,†,‡^	0.28 ± 1.35^§,‖^	0.12 ± 1.10^¶^	−0.13 ± 1.29^*,†,‡^	0.15 ± 1.77^§,‖^	0.03 ± 1.57	0.45 ± 1.70^*,†,‡^	−0.06 ± 1.50^§^	−0.36 ± 1.48

*Note*: Radiographic ridge width change measured at 3‐, 5‐ and 7‐mm from alveolar crest expressed in mm (mean ± *SD*). ^*,†,‡,§,‖,¶^: Similar superscript within a given column indicates a statistically significant difference (*p* < 0.05) between the groups at that measurement point.

### 3.2 Dimensional change analysis

Pearson correlation analysis showed no significant correlation between lingual plate thickness and ridge width change. For the buccal plate thickness, there was no significant correlation on the distal root. However, there was a small but significant negative correlation on the mesial root between buccal plate thickness at 3 mm and ridge width change at 3 mm (*R* = 0.25, *p* = 0.03), and between buccal plate thickness at 5 mm and ridge width change at 5 mm (*R* = 0.34, *p* = 0.003), indicating greater loss of ridge width when a thinner buccal plate was present.

### 3.3 Radiographic measurement reproducibility

Overall, inter‐examiner agreement of digital radiographic measurements was determined by 52 repeated measurements performed by three primary examiners (A.M.A, S.A., C.W.). The interclass correlation, mean ICC = 0.95, indicated excellent reproducibility between examiners. Intra‐examiner reliability was determined by 51 repeated measurements performed by a single examiner (A.M.A.), with mean ICC = 0.88.

### 3.4 Histomorphometric analysis

Histologic comparison of wound healing among the groups showed significantly greater vital bone formation (62.10%) in the dPTFE membrane alone group (test 3) than the other groups (*p* < .05; Table 6). There was no significant difference in vital bone formation among the control, test 1, and test 2 groups.

**TABLE 6:** Histologic outcomes

Group		%Vital bone (mean ± *SD*)	%Residual graft (mean ± *SD*)	%CT/other (mean ± *SD*)
Walker et al. (2017)	Control (no RP)	37.97% ± 12.0^a^	N/A	*62.43% ± 12.5^‖,¶^
	Test 1 (FDBA+ dPTFE)	25.41% ± 17.8^†^	10.58% ± 7.1^§^	*64.01% ± 13.4^‖,¶^
Al Harthi et al. (2019)	Test 2 (FDBA + CWD)	30.43% ± 21.4^‡^	23.22% ± 15.2^§^	46.35% ± 11.5^‖^
Present study	Test 3 (dPTFE alone)	62.10% ± 31.5^*,†,‡^	N/A	37.94% ± 31.5^¶^

*Note*: No bone graft was used in control and test 3 groups. ^a,†,‡,§,‖,¶^: Similar superscript within a given column indicates a statistically significant difference (*p* < 0.05) between the groups for that histologic parameter.

* No significant difference in CT/other between control and test 1 groups.

## 4 DISCUSSION

RP procedures have been shown to reduce vertical and horizontal alterations of the alveolar ridge post‐extraction (Ten Heggeler et al., 2011; Vignoletti et al., 2012). Other studies (Hoffmann et al., 2008; Laurito et al., 2016), along with the present study, have demonstrated clinical effectiveness of the use of dPTFE in RP procedures when the membrane is left to heal exposed to the oral cavity. dPTFE membrane acts as an occlusive barrier preventing epithelial migration into the socket. Furthermore, it has been shown to prevent bacterial penetration due to the disparity between the average size of bacteria (1–2 μm) and the membrane's porosity size of less than 3 μm, with low risk of infection when exposed to oral cavity (Carbonell et al., 2014).

When assessing the radiographic dimensional changes, results of this study showed 1.48, 1.05, and 1.0 mm less vertical bone loss compared to the control group at the midbuccal aspect of the socket for test 1, test 2, and test 3, respectively. This is consistent with the vertical dimension alteration following RP reported in a recent systematic review (Avila‐Ortiz et al., 2014).

There was significantly less ridge width reduction in the dPTFE membrane alone group (Test 3) than in the other groups at most measurement points 3 and 5 mm apical to the bony crest. This could be explained by the significant difference in baseline buccal plate thickness between the groups, as the dPTFE membrane alone group (test 3) had thicker buccal bone measurements than the other groups. Findings of the current study indicated a significant negative correlation between buccal plate thickness and ridge width reduction. This is in agreement with studies that found dimensional changes in both width and height to be greater at sockets with <1 mm buccal bone thickness (Avila‐Ortiz et al., 2019; Cardaropoli et al., 2014). Although there was no statistically significant difference in ridge width change between the natural healing (control), FDBA with dPTFE (test 1) and FDBA with CWD (test 2) groups in the current study, the no RP group (control) had consistently more buccal bone loss than any of the other RP techniques.

A recent case report described the periosteal inhibition technique by the placement of dPTFE membrane between the periosteum and the buccal bone of an extraction socket, resulting in stable ridge dimensions (Nguyen et al., 2019). The membrane creates a space allowing the formation of blood clot which acts as a matrix for bone formation (Buser et al., 1994). It is unknown why, in the current study, there was less reduction in ridge width in the test 3 group in which no bone graft was used with the dPTFE membrane compared to the test 1 group in which a bone graft was used with the dPTFE membrane. The difference in buccal plate thickness between the groups may provide a partial, although incomplete explanation, as the buccal plate thickness in the test 3 group was 0.7–1.5 mm greater than the test 1 group at the various measurement points (Table 3).

Relative to histologic outcomes, the test 3 group, in which no bone graft was used, had the highest percentage of vital bone formation among the groups at the 3‐month time point. Early in healing after RP, implanted bone graft material, such as that used in test 1 and test 2 groups, has not completely turned over. As time progresses, vital bone replaces implanted graft material (Nelson & Mealey, 2020; Whetman & Mealey, 2016). It is likely that over longer healing periods the replacement of allograft by vital bone in test 2 group would result in percentage of vital bone closer to that of test 3 group. The significant difference in the percentage of residual graft between test 1 and test 2 may be attributed to differences in inductivity of the bone graft between tissue donors (Schwartz et al., 1998).

Bone grafting at time of implant placement due to exposure of a small amount of implant surface was needed in 25% of cases in the control group compared to 10% in test 1 group, 0% in test 2 group and 16% in test 3 group (Al Harthi et al., 2019; Walker et al., 2017). Therefore, the percentage of cases where implant placement was completed without the need for additional grafting was higher in RP groups compared to the control group. Nevertheless, subjects in all groups were able to achieve the end goal of restoratively driven implant placement with good primary stability.

The results of this study should be interpreted with caution. The baseline buccal plate thickness in test 3 group was significantly greater than the other groups. It is possible that the minimal change in ridge width seen in test 3 group was due to residual bone thickness following extraction rather than the type of RP technique utilized and therefore may consider a limitation of this study. The effect of dPTFE membrane alone as RP material in areas with different buccal bone thickness warrant further investigation.

## 5 CONCLUSION

The present data suggest that without RP there was consistently more buccal bone loss than with any of the RP techniques. The use of dPTFE membrane alone for RP at molar extraction sites showed excellent outcomes in terms of reducing ridge dimensional changes and formation of vital bone in the former socket, and therefore should be considered as a viable treatment option for RP procedures at molar sites.

## CONFLICT OF INTEREST

All authors report no conflicts of interest

## FUNDING INFORMATION

No funding was provided for this study. The authors thank Osteogenics Biomedical (Lubbock TX, USA) for providing the materials for this study.

## AUTHOR CONTRIBUTIONS

Arwa Al Hugail, Brian Mealey, Christopher Walker, Shaimaa Al Harthi, Mylinh Duong, David Lasho, and Guy Huynh‐Ba were all involved in study design, patient management, data collection, and writing and revising the manuscript. Thomas Prihoda was involved in statistical analysis and interpretation. All authors approved the version to be published and agreed to be accountable for all aspects of the work.

## Data Availability

The data that support the findings of this study are available from the corresponding author upon reasonable request.
